# BDNF SNP C270T Modifies the Association Between History of Head Injury and Cognitive Status in Older Adults

**DOI:** 10.1093/geroni/igab046.2500

**Published:** 2021-12-17

**Authors:** Mikaela Drewel, Hector Gonzalez, Gail Rattinger, Joshua Matyi, Alexandra Hammond, Kaitlyn Kauzor, Mona Buhusi, JoAnn Tschanz

**Affiliations:** 1 Utah State University, Logan, Utah, United States; 2 Binghamton University, Binghamton University, New York, United States

## Abstract

Brain derived neurotrophic factor (BDNF), is a neurotrophin involved in neurogenesis and neuroplasticity. Several BDNF genes have been associated with cognitive function. Studies suggest head injury (HI) alters BDNF levels, and activities enhancing BDNF signaling promote better cognitive outcomes. We investigated the relationship between HI and BDNF single-nucleotide polymorphisms (SNPs) in predicting cognitive performance in a population-based sample of older adults. 4165 participants (56.7% female), dementia-free at baseline, were assessed triennially [follow-up years: mean (SD) = 5.85 (4.20), median = 7.33, maximum = 11.39]. Mean (SD) age was 75.36 (6.84). Cognition was assessed using the Modified Mini-Mental State Exam (3MS) and HI history from self-report. We examined interactions between BDNF SNPs [rs56164415 (BDNF C270T), rs6265 (Val66Met), rs2289656 (BDNF receptor trkB), and rs2072446 (NGF/BDNF receptor p75)] and history of HI (none, one, or multiple) in predicting cognitive decline. Covariates included age, education, sex, and apolipoprotein (APOE) E4 allele presence. Linear mixed-effect models indicated BDNF C270T significantly modified the association between HI and cognitive status (p < .006). Specifically, minor T allele carriers with single or multiple HI scored on average 2.08 and 3.21 points lower on the 3MS, respectively, than non-T carriers with no HI. Unexpectedly, there was a trend for APOE4*HI (p = .078) in that APOE E4 carriers with multiple HI scored higher than those lacking APOE E4 and HI. In this population-based sample, rs56164415 predicted cognitive outcomes that varied by history of HI. Factors influencing BDNF signaling may provide a potential avenue for intervention in recovery from HI.

